# Manipulating Magnetic Damping of Fe/GeTe Heterostructures by Band Engineering

**DOI:** 10.1002/advs.202411798

**Published:** 2024-12-18

**Authors:** Xu Yang, Jia‐Wan Li, Yan Li, Liang Qiu, Hao‐Pu Xue, Jin Tang, Hai‐Feng Du, Rui Sun, Qing‐Lin Yang, Jia‐Nan Liu, Xiang‐Qun Zhang, Wei He, Yusheng Hou, Zhao‐Hua Cheng

**Affiliations:** ^1^ Beijing National Laboratory for Condensed Matter Physics Institute of Physics Chinese Academy of Sciences Beijing 100190 China; ^2^ Songshan Lake Materials Laboratory Dongguan Guangdong 523808 China; ^3^ Guangdong Provincial Key Laboratory of Magnetoelectric Physics and Devices Center for Neutron Science and Technology School of Physics Sun Yat‐Sen University Guangzhou 510275 China; ^4^ School of Physical Sciences University of Chinese Academy of Sciences Beijing 100049 China; ^5^ Anhui Province Key Laboratory of Condensed Matter Physics at Extreme Conditions High Magnetic Field Laboratory of the Chinese Academy of Sciences University of Science and Technology of China Hefei 230031 China; ^6^ Institute of Physical Science and Information Technology Anhui University Hefei 230601 China

**Keywords:** band engineering, hybridized band structures, magnetic damping, magnetic dynamics, Rashba effect

## Abstract

Understanding and manipulating magnetic damping, particularly in magnetic heterostructures, is crucial for fundamental research, versatile engineering, and optimization. Although magnetic damping can be enhanced by the band hybridization between ferromagnetic and nonmagnetic materials at the interface, the contribution of individual subbands on the hybridized bands to magnetic damping is fully unexplored. Here, it is found that magnetic damping α_eff_ is modified by the Fermi level in Fe/GeTe heterostructures via Bi doping. By combining angle‐resolved photoemission spectroscopy and density functional theory calculations, the enhancement of damping originated from the strongly hybridized band structures between Fe and the surface Rashba bands of GeTe are unveiled. More interestingly, the Fermi level modulates the density of states (DOS) ratio between the subbands of GeTe and the total DOS of hybridized states, which is directly proportional to the magnetic damping. This work gives an insightful physical understanding of the magnetic damping influenced by the hybridized band structures and opens a novel avenue to manipulate magnetic damping by band engineering.

## Graphical abstract

This study finds enhanced and tunable magnetic damping in the Fe/GeTe heterostructure by changing the Fermi level, and the magnitude of damping is related to the density of the state of the hybridized band structures. This paper proposes the possibility of controlling the magnitude of magnetic damping by band engineering.

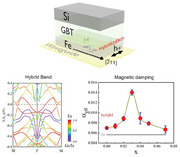

## 1 Introduction

Gilbert damping characterizes the angular momentum transfer from spin to other degrees of freedom. Prior theoretical and experimental studies have demonstrated that the local Gilbert damping in ferromagnets can be influenced by spin‐orbit coupling, the density of states (DOS) at the Fermi surface, and the momentum scattering time.^[^ ^1^ ^]^ In ferromagnet (FM)/nonmagnetic (NM) heterostructures, which are the building blocks of spintronic devices, non‐local Gilbert damping arises due to spin pumping.^[^ ^2^ ^]^ Effective control of this non‐local damping can significantly enhance spintronic device performance by optimizing control times and reducing critical current densities for magnetization switching.^[^ ^3^ ^]^ Despite its importance, the precise mechanisms for controlling non‐local damping remain inadequately understood.

The Fermi level in topological materials significantly influences non‐local damping in YIG/(Bi_x_Sb_1‐x_)_2_Te_3_ heterostructures by controlling the weight between surface and bulk states.^[^ ^2a^ ^]^ More recently, GeTe, as one of the Ferroelectric Rashba semiconductors (FERSCs), has the largest Rashba splitting size and induced a lot of novel phenomena in spintronics and spin dynamics, such as the room temperature nonreciprocal transport,^[^ ^4^ ^]^ the room temperature ferroelectric switching spin‐charge conversion,^[^ ^5^ ^]^ and the anisotropic nonlocal damping.^[^ ^6^ ^]^ Besides the bulk Rashba bands, the surface states (SS) of GeTe also exhibit Rashba band characteristics.^[^ ^7^ ^]^ Given the volatile Rashba band structure of GeTe depending on the adjacent materials and the magnetism at the interfaces,^[^ ^8^ ^]^ it is highly desirable to investigate the response of magnetic damping to the relative position of the band structure with the Fermi level after depositing the magnetic materials. According to our previous work,^[^ ^8^ ^]^ the surface states of GeTe hybridized with the band structures of Fe, which is a system to study the influence of the hybridized state on magnetic damping. Although band hybridization‐enhanced magnetic damping has been studied in Fe/Bi_2_Se_3_ heterostructure,^[^ ^9^ ^]^ how to manipulate the magnitude of the magnetic damping by hybridized states is unclear. This topic is important not only for spintronics but also fundamental for physics.

Here, we realized non‐monotonic magnetic damping of Fe/(Ge_1‐x_Bi_x_)Te (denoted as Fe/GBT) (x = 0.00 ‐ 0.07) bilayers verified by Ferromagnetic resonance (FMR) measurements and density functional theory (DFT) calculations. Combining the angle‐resolved photoemission spectroscopy (ARPES) of GBT and DFT calculations, we find that the increase of Bi concentration mainly shifts the Fermi level without changing the Rashba splitting size. Since the bulk states move to the deeper binding energy, one can expect that the spin‐pumping effect should induce a decreased magnetic damping with Bi‐doping. In contrast to our prediction, we observe that the magnetic damping shows a maximum value at x = 0.03. Our DFT calculations suggest that this enhanced and non‐monotonic magnetic damping originates from the strong hybridizations between the bands of Fe and the surface Rashba bands of GeTe, and the Fermi level modulates the α_eff_ of Fe/GBT through tuning the DOS ratio between GeTe subbands and the total DOS of the hybridized states. Our work provides an insightful understanding of magnetic damping induced by the hybridized band structures.

## 2 Results and Discussion

**Figure 1:**
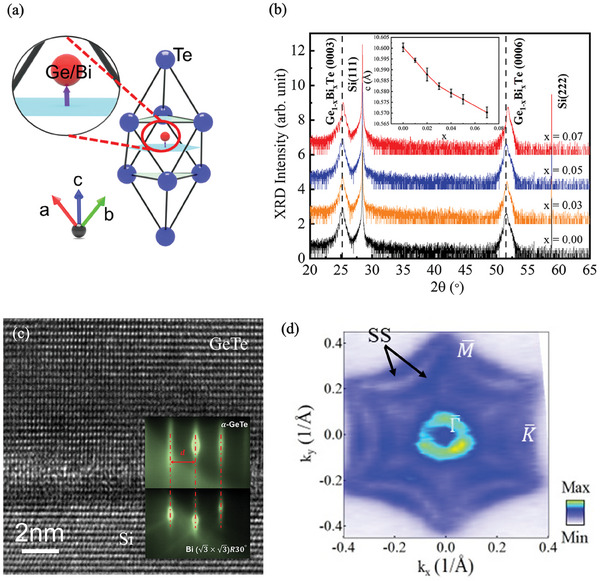
Unit cell and the band structure of Ge_1‐x_Bi_x_Te. a) The unit cell of GeTe with Bi doping. The light‐green coloring represents two planes made up of Ge/Bi atoms. The light‐blue plane crosses the center of GBT and is perpendicular to [0001], and the purple arrow represents the electric polarization. b) XRD patterns of GBT with different Bi concentrations. The insert shows the lattice constant *c* of GBT as a function of Bi concentration. c) High‐angle annular dark field image of GeTe. The insert is the RHEED patterns of Bi/Si and GeTe. d) The Fermi surface of GeTe.

Previous studies show that the doping of Bi atoms in α‐GeTe gains a super‐high thermoelectric figure‐of‐merit and increases the resistance as well.^[^ ^10^ ^]^ It was proved that Bi atoms can donate electrons to GeTe and change its Fermi level. Therefore, we choose Bi as the doping element. **Figure** **1**a represents the unit cell of Bi‐doped GeTe. Figure 1b demonstrates the x‐ray diffraction (XRD) patterns of (Ge_1‐x_Bi_x_)Te (x = 0.00, 0.03, 0.05, and 0.07). The positions of (0003) and (0006) of GBT diffraction peaks shift to the higher diffraction angle, suggesting that the lattice constant *c* decreases with increasing Bi concentration (see the inset of Figure 1b). No impurity phase is detected in the thin films. To further verify the quality of the GeTe thin films, we perform a high‐angle annular dark field (HAADF) image of a ($11\bar{2}0$) cross‐section of the GeTe/Si(111) and Ge_0.93_Bi_0.07_Te/Si(111) thin film, as shown in Figure 1c and Figure S2 (Supporting Information). The perfect atom arrangement illustrates the high‐quality nature of our thin films and the Bi atoms don't form the clusters. Figure 1d illustrates the Fermi surface of GeTe measured by the in situ ARPES. In good agreement with previous studies,^[^ ^6^, ^11^ ^]^ the bulk states are the coexisting Rashba and Dressehaulls states, and the surface states are the Rashba states.

**Figure 2:**
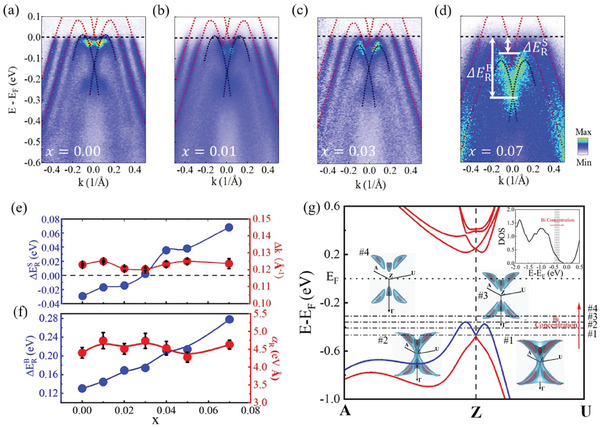
Band structure of GBT along $\bar{K}-\bar{\Gamma }-\bar{K}$. a–d) The band structure of Ge_1‐x_Bi_x_Te with different Bi concentrations. The black and red dashed lines are the fitting results of bulk and surface states. e,f) energy shifting of surface Rashba cross point (e) and bulk Rashba cross point (f). The right axis is the surface Rashba splitting size (e) and bulk Rashba parameters (f) changing with Bi concentration. g) DFT calculation of the bulk states of GeTe. The dot‐dashed lines signed by #1 – #4 represented the tunable Fermi level by Bi atoms. The inset pictures illustrate the 3D isoenergetic surfaces for four Fermi levels (#1 – #4). The top right corner is the DOS of the bulk states of GeTe.

Before studying the spin dynamics influenced by the different Fermi levels, we measured the dependence of ARPES spectra on the Bi concentration. **Figure** **2**a–d show the ARPES results ($\bar{M}-\bar{\Gamma }-\bar{M}$) of GBT with x = 0.00, 0.01, 0.03, and 0.07 (others shown in Figure S3, Supporting Information). With increasing the Bi concentration, the bands rigidly shift toward higher binding energies, as shown in Figure 2e,f. We cut the energy distribution curves (EDCs) to estimate the energy shift and fit the Rashba parameters (α_*R*_) of bulk states by $E_{\pm }=\left(\frac{\hbar^{2}k^{2}}{2m^{*}}\pm \left|\alpha_{R}\right|k+E_{0}\right)$ (Where m* is the effective mass of carriers, and E_0_ is the energy of the band edge.) and the splitting size (Δ*k_s_*) of surface Rashba states, by taking the k=0.31 Å^−1^ that is the Fermi wave vector of the undoped sample. Then we estimated the magnitude of the energy band shifting due to Bi doping. The shifting of the surface states is defined as $\Delta E_{R}^{S}=E_{F}\;-E_{R}^{S}$, where $E_{R}^{S}$ is the surface Rashba crossing point. Similarly, the shifting of bulk bands is defined as $\Delta E_{R}^{B}=E_{F}\;-E_{R}^{B}$, where $E_{R}^{B}$ is the bulk Rashba crossing point. From Figure 2e,f, the nearly identical Rashba parameter and splitting size imply that the Bi doping does not change the Rashba states of GeTe, but only shifts the Fermi level. This behavior is different from the K dope of GeTe.^[^ ^7d^ ^]^ Although doping Bi and K atoms can modify the Fermi level and can observe the whole dispersion of the surface states of GeTe, the difference is doping K atoms at the surface can shrink the split size of the surface Rashba states.^[^ ^7d^ ^]^ Due to the inner potential *V*_inner_ =  15 eV, ^[^ ^7a^ ^]^ the *k*_z_ of these ARPES spectra is Z+0.14 Å^−1^, where Z is the edge of Brillouin Zone (BZ) along *k*_z_.^[^ ^7a^ ^]^ From the ARPES spectra, both bulk states and surface states cross through the Fermi surface when x  <  0.04. When x ≥ 0.04, only surface states cross over the Fermi level. Since only one *k_Z_* cannot evaluate the situation of the whole BZ, we combined it with DFT calculation to determine the change of the band structures after doping Bi atoms. Figure 2g plots the bulk bands of GeTe near the *Z* point. Because a model of perfect GeTe crystal without Ge vacancies is built during DFT calculation, its Fermi level is higher than the experimental one. The dot‐dashed lines signed by #1–#4 in Figure 2g are the cutting lines, implying the Fermi level changes by increasing the Bi concentration. The insert figures signed by #1– 4 illustrate the isoenergetic surfaces of the bulk Rashba bands, and the volume surrounded by these isoenergetic surfaces is shrunk when elevating the Fermi level. This implies the density of states (DOS) of the bulk Rashba states in the whole BZ is decreased, as shown in the inset figure at the top right corner of Figure 2g, i.e., when Bi atoms are heavily doped, the Fermi level is far away from bulk Rashba states of GeTe.

**Figure 3:**
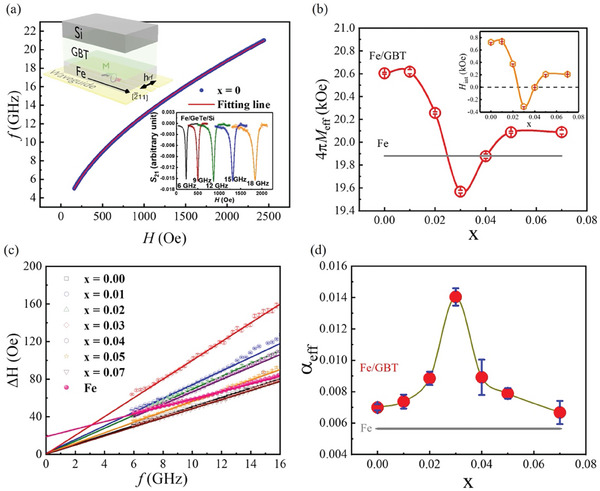
FMR results of Fe/GBT heterostructures. a) The Kittel model of Fe/GBT. The left‐top corner figure is the FMR configuration of the Fe/α‐GeTe bilayer. The insert figure shows the FMR ReS21 signal of Fe/GeTe bilayer for different selected frequencies (6, 9,12,15,18 GHz) at room temperature. b) The Bi concentration dependence of 4πM_eff_ of Fe/GBT and the pure Fe thin film. The inset of (b) shows the Bi concentration dependence of H_int_. c) The relationship between resonant linewidth ΔH and resonant frequency *f* for different doping concentrations. d) The Bi concentration dependence of effective damping of Fe/GBT, and the grey line is the damping of pure Fe thin film.

To figure out the evolution of damping of Fe/GBT with Bi concentration, a Fe thin layer with a thickness of 12 nm was epitaxially grown on GBT. The sample qualities of Fe/GBT are described in Supplemental Information Figure S1 (Supporting Information). To investigate the spin dynamics of Fe/GBT by FMR measurements, a magnetic field was applied along Fe [$\bar{2}11$], as illustrated in the insert of **Figure** **3**a. Figure 3a shows the relationship between resonance frequency and magnetic field of Fe/GeTe, which is extracted from the FMR ReS_21_ signal fitted by the Lorentzian function.^[^ ^2^, ^12^ ^]^ The right bottom corner insert of Figure 3a is the representative transmission data of ReS_21_ of Fe/GeTe/Si at room temperature. It displays the FMR absorption with several frequencies (𝑓 = 6, 9, 12, 15, and 18 GHz). By comparing the resonant field of Fe/GBT and Fe at 12 GHz, we found that the resonance field of Fe/GBT can be modified by the Bi concentration (shown in Figure S4, Supporting Information). Therefore, we fitted the *f–*H curves by the following Kittel equation (details in Figure S4, Supporting Information) adding a phenomenological effective field H_eff_, and the effective anisotropy field 4πM_eff_,^[^ ^2b^ ^]^

(1) $$f=\frac{\gamma }{2\pi }\sqrt{\left(H_{\mathrm{res}}+H_{\mathrm{eff}}\right)\left(H_{\mathrm{res}}+H_{\mathrm{eff}}+4\pi M_{\mathrm{eff}}\right)}$$

where γ is the gyromagnetic ratio, H_res_ is the resonance field. The effective anisotropy field 4πM_eff_ of Fe/GBT is defined as 4π M_eff_ =  4πM_s_ + H_int_, where 4πM_s_ is demagnetization field of Fe, and H_int_ is an interfacial anisotropy field induced by Fe and GBT. Figure 3b shows the 4πM_eff_ as a function of Bi concentration. The 4πM_eff_ decreases until x = 0.03 and then increases with increasing Bi doping concentration x. This quite different behavior with Fe thin film implies an interfacial coupling between Fe and GeTe. The insert of Figure 3b shows the Bi concentration dependence of H_int_, which has a similar behavior with 4πM_eff_. The similar dependences of 4πM_eff_, and H_int_ on Bi concentration implies the interfacial coupling between the Fe and GeTe can be modified by the Fermi level.

Figure 3c shows the relationship between the half‐width of maximum (HWHM) of the ReS_21_ signal, ΔH, and frequency *f* for different Bi doping concentrations. The effective magnetic damping α_eff_ is extracted from the fitting of the relation between Δ*H* and *f* (detailed in the S4, Supporting Information). By extracting the extrinsic factor of the damping (detailed in the Figure S4, Supporting Information), the α_eff_ of Fe/GBT bilayer increases and reaches a maximum value at *x*  =  0.03, in which the Fermi level passes through the surface Rashba crossing point,^[^ ^13^ ^]^ then the damping decreases with further increasing Bi concentration, as shown in Figure 3d. We find that the behavior of α_eff_ with Bi concentration identical to that of H_res_ (shown in Figure S5, Supporting Information), which implies the interfacial coupling in Fe/GBT play an important role in magnetic damping. However, the Bi element has a strong SOC, but our conclusion is that the SOC of Bi atoms is not influenced by the results. There are two reasons: 1) if Bi atoms change evidently the SOC of the GeTe, the Rashba splitting size should be increased by increasing the Bi concentration. However, the ARPES results of our work show that the Rashba splitting size is nearly the same for all the GBT samples shown in Figure 2a–f. 2) If the Bi atoms increase the SOC of the system, the damping should be increased by increasing the Bi concentration because the damping is related to the strength of SOC. Since the bulk states are far away from the Fermi level gradually with increasing the Bi concentration, the damping should be expected to decrease because of weakening the spin‐pumping effect. However, the experimental results are different from the expected ones. Combined with the unchanged Rashba parameters of different Bi concentration samples, the SOC of Bi atoms is not the key factor to influence the damping. Therefore, we believe that the strong interfacial coupling between Fe and GeTe can be tuned by the Bi concentration, which modifies the damping of the Fe/GeTe heterostructures.

**Figure 4:**
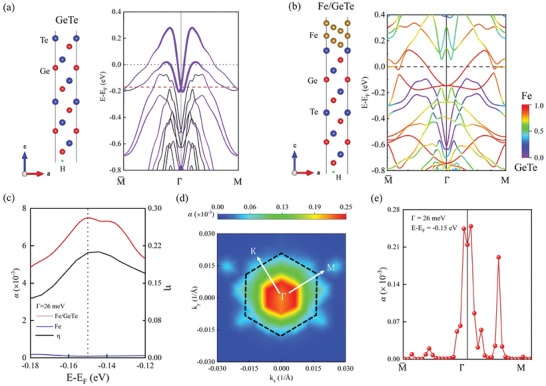
DFT calculated band structures and damping of Fe/GeTe heterostructure. a) Atomic models and band structure of GeTe thin film. The weight of the top two layers is indicated by purple dots. b) Atomic models and band structure of Fe/GeTe. The colors represent the value of the hybridized ratio. The purple represents the GeTe subbands, the red represents the Fe subbands, and the other colors represent the hybridized states. c) The Fermi level dependence of the calculated Gilbert damping of Fe/GeTe and Fe thin film. The black line is the DOS ratio between the GeTe subbands and the total DOS of hybridized states at different binding energies. d) The first BZ distribution of *k*‐dependent contributions to Gilbert damping of Fe/GeTe at E_B_  =   − 0.15 eV. The DOS is calculated in the hexagonal region signed by the black dashed lines. e) same as (d) but for the high‐symmetry *k*‐paths.

We perform DFT calculations to investigate the band structures and damping of the Fe/GeTe heterostructure. Since Bi concentration in GBT mainly tunes the position of the Fermi level, we build the Fe/GeTe bilayer and shift its Fermi level within the rigid band model to gain insight into α_eff_ of Fe/GBT. By comparing the atom‐resolved band structure of the free‐standing GeTe film and Fe/GeTe shown in **Figure** **4**a,b, we see that the surface Rashba bands of GeTe are strongly depressed by the presence of Fe thin film; explicitly, one part of surface Rashba bands is flipped over Fermi level while the other part is moved down to near E_B_ =  E −  E_F_ =   − 0.2 eV. This indicates the strong hybridization between Fe and GeTe thin films, consistent with the previous study.^[^ ^8^ ^]^ Due to the hybridization, the damping of Fe/GeTe is strongly enhanced, and it reaches the maximum when the Fermi level passes through the surface Rashba crossing point (i.e., around E_B_ =   −0.15 eV, corresponding to the case of x = 0.03) as shown in Figure 4c. By examining the *k*‐dependent contributions to Gilbert damping shown in Figure 4d,e, we see that the bands of Fe hybridize strongly with the bands of GeTe in the vicinity of Γ point, leading to the largest contribution to the damping parameter. Compared with the damping of pure Fe film (Figure 4c), we conclude that the enhanced damping of Fe/GBT is determined by the strong hybridizations between interfacial bands of Fe and GeTe.

Based on the scattering theory,^[^ ^14^ ^]^ the Gilbert damping can be determined by the following equation:^[^ ^9^ ^]^

(2) $$\begin{array}{ccc} \alpha_{\mu \nu } & = & -\frac{\pi \hbar \gamma }{M_{S}}\sum_{\mathrm{ij}}\langle \psi_{i}\left|\frac{\partial H}{\partial u_{\mu }}\right|\psi_{j}\rangle \langle \psi_{j}\left|\frac{\partial H}{\partial u_{\nu }}\right|\psi_{i}\rangle \\ & & \times \delta \left(E_{F}-E_{i}\right)\delta \left(E_{F}-E_{j}\right) \end{array}$$

here, *E_F_* is the Fermi level, and *u* is the deviation of a normalized magnetic moment away from its equilibrium, that is, *m*  = *m*_0_  + *u* with *m*_0_  = *M*_0_ /*M_S_*. From the delta function δ(*E_F_* − *E_i_*)δ(*E_F_* − *E_j_*) in Equation (2), one can see that only the hybridized bands at or close to the Fermi level have a dominant influence on the Gilbert damping. In our calculations, there is no spin‐pumping effect. Then we investigate the distributions of the *k*‐dependent contributions to Gilbert damping in the first BZ to unveil how the Fermi level modulates α_eff_ of Fe/GeTe. By comparing Figure 4d and Figure S8 (Supporting Information), we observed a significant difference between the distributions of the *k*‐dependent contributions to Gilbert damping when the Fermi level goes from − 0.18 to − 0.12 eV. In particular, when the Fermi level is − 0.15 eV, the contributions to Gilbert damping are almost from the *k* points in the vicinity of the Γ point where the surface Rashba state appears in the free‐standing GeTe film. In contrast, when the Fermi level deviates from − 0.15 eV, large contributions to Gilbert damping can come from random *k* points in the first BZ. Comparing these random k points with the band structures, all the bands at these random *k* points are the hybridized bands. To determine what physical parameter influenced the dampings, we calculated the DOS of the hexagonal region shown in Figure 4d. The results show that the ratio between the DOS of GeTe spectrum weight and the total DOS, $\eta =\frac{N_{\mathrm{GeTe}}}{N_{\mathrm{Fe}}+N_{\mathrm{GeTe}}}\;$, is strongly related to the damping behavior at the Fermi level (the results of other regions and other ratios are shown in Figure S9, Supporting Information). Therefore, we conclude that such distinctly different dependence of the contributions to Gilbert damping on the Fermi level suggests that the Fermi level modulates the α_eff_ of Fe/GBT through tuning the hybridization DOS between the spectrum weight of GeTe and the total DOS of the hybridized bands.

## 3 Conclusion

In summary, we find enhanced and tunable magnetic damping in the Fe/GeTe heterostructure by changing the Fermi level. With the help of DFT calculations, we find that the enhanced damping compared with the Fe thin film originates from the hybridized band structures between Fe and the surface Rashba states of GeTe. Doping Bi atoms to change the Fermi level of GeTe modifies the hybridization of the Fe subbands in the hybridized states. Besides that, the Fermi level modulates the α_eff_ of Fe/GBT by tuning the hybridization DOS between the subbands of GeTe and the total DOS of the hybridized bands. Our work opens the possibility that the surface states of GeTe can modify the damping by interlayer hybridization with the magnetic layer and gives an insightful understanding of the influence of band hybridization on magnetic damping, which paves a new way to control the magnitude of magnetic damping by band engineering.

## 4 Experimental Section

### Sample Fabrications

Ge_1‐x_Bi_x_Te thin films were grown in an ultrahigh vacuum chamber (base pressure: 1.0 × 10^−10^mbar). Before growing Ge_1‐x_Bi_x_Te, one bilayer Bi_2_ was first grown on Si (111) as a seeding layer to decrease the mismatch between Si (111) and Ge_1‐x_Bi_x_Te. The Ge_1‐x_Bi_x_Te thin films were grown by evaporation of high purity Ge (99.99%) and Te (99.999%) from Knudsen cells with the flux of ≈1:10 and high purity Bi (99.999%) via electron beam evaporation onto the Si (111) substrates. The ratio of Bi atoms was determined by the flux ratio between Bi and Ge. The temperatures of the Ge source, Te source, and the substrate were kept at 1160, 310, and 140 °C, respectively, annealed to 100 °C for 1 h. After ARPES performance, 12 nm Fe was grown on GBT thin films via MBE. The growth rates determined by ex situ small angle X‐ray reflectivity (XRR) were 0.73 and 0.05 nm min^−1^ for GBT and Fe, respectively.

### ARPES Measurements

To perform ARPES measurements, all GBT thin films were fabricated and then transported to the in situ ARPES. ARPES measurements were performed at room temperature with a He discharge lamp with a photon energy of 21.2 eV as the photon source and Scienta DA30‐L as the electron energy analyzer. The energy and momentum resolution was better than 20 meV and $0.01{\overset{\circ }{A}}^{-1}$, respectively.

### DFT Calculations

DFT calculations were performed using the Vienna ab initio Simulation Package (VASP) at the level of the generalized gradient approximation.^[^ ^15^ ^]^ The projector augmented wave pseudopotentials^[^ ^16^ ^]^ and a plane‐wave cutoff energy of 400 eV was utitlized. The α‐GeTe (111) substrate was simulated by twelve layers of atoms, with an in‐plane lattice constant of 4.233 Å. In the structural optimization of the Fe (111)/*α*‐GeTe heterostructure, the positions of all atoms except those of the second six‐layer atoms of *α*‐GeTe were fully relaxed until the magnitude of the force on each atom was less than 0.01 eV Å^−1^. The Te‐terminated surface of α‐GeTe was passivated by hydrogen atoms. The van der Waals (vdW) correction in the form of the semi‐empirical DFT‐D3 method^[^ ^17^ ^]^ was adopted in the calculations of Fe (111)/α‐GeTe heterostructure.

### Gilbert Damping Calculations

The Gilbert damping constant, α, was acquired by extending the torque method for the study of magnetocrystalline anisotropy.^[^ ^9^ ^]^ To ensure the numerical convergence, the first BZ of Fe/α‐GeTe was sampled by a 37 × 37 × 1 Γ‐centered grid and the number of bands for the second‐variation step was set to 240.

### FMR Measurements

Vector Network Analyzer ferromagnetic resonance (VNA‐FMR) measurements were performed by placing the sample face‐down on a co‐planar waveguide (CPW) and recording the transmission coefficient S_21_ at room temperature.

### TEM Measurements

The GBT samples for TEM measurements were fabricated by a focused ion beam and scanning electron microscopy dual beam system (Helios NanoLab 600i, FEI).^[^ ^18^ ^]^ The high‐resolution crystal structure was conducted in a TEM (Talos F200X, FEI) operated at 200 kV.

## Conflict of Interest

The authors declare no conflict of interest.

## Author Contributions

X.Y. and J.‐W.L. contributed equally to this work. Z.H.C. is responsible for the project and conceived this study and the experiments. X.Y. grew the samples and performed ARPES and FMR measurements. J. W. L., L. Q., and Y. S. H. carried out DFT and damping calculations. J.T. and H.F. performed HRTEM measurements. Y.L., H.P.X. R. S., Q.L. Y., J.N., X.Q. Z., and W.H. help to measure x‐ray diffraction, FMR, ARPES, and magnetotransport measurements. X.Q.Z. and W.H. contributed greatly to the setup of MBE and ARPES. Z.H.C. X.Y and Y.S.H. wrote the paper. All authors discussed the results and worked on data analysis and manuscript preparation. The authors thank Profs. Qing‐Feng Zhan, Dali Sun, and Hao‐Liang Liu for their critical reading and constructive suggestions.

## Supporting information



Supporting Information

## Data Availability

The data that support the findings of this study are available from the corresponding author upon reasonable request.
