# Supplementary material for: Manipulating Magnetic Damping of Fe/GeTe Heterostructures by Band Engineering
Source: Adv Sci (Weinh). 2024 Dec 18;12(6):2411798. doi: 10.1002/advs.202411798 (PMC11809334; doi:10.1002/advs.202411798)
Supplement: Supplementary file 1 — Supporting Information [file ADVS-12-2411798-s001.docx]

**Supporting Information for**

**Manipulating Magnetic Damping of Fe/GeTe Heterostructures by Band Engineering**

Xu Yang^1,6,7^, Jia-Wan Li^2,7^, Yan Li^1^, Liang Qiu^2^, Hao-Pu Xue^1,3^, Jin Tang^4,5^, Hai-Feng Du^4,5^, Rui Sun^1,3^, Qing-Lin Yang^1,3^, Jia-Nan Liu^1,3^, Xiang–Qun Zhang^1^, Wei He^1^, Yusheng Hou^2,*^ and Zhao-Hua Cheng^1,3,6,*^

^1^Beijing National Laboratory for Condensed Matter Physics, Institute of Physics, Chinese Academy of Sciences, Beijing 100190, China;

^2^ Guangdong Provincial Key Laboratory of Magnetoelectric Physics and Devices, Center for Neutron Science and Technology, School of Physics, Sun Yat-Sen University, Guangzhou, 510275, China;

^3^School of Physical Sciences, University of Chinese Academy of Sciences, Beijing 100049, China;

^4^Anhui Province Key Laboratory of Condensed Matter Physics at Extreme Conditions, High Magnetic Field Laboratory of the Chinese Academy of Sciences, and University of Science and Technology of China, Hefei 230031, China;

^5^Institute of Physical Science and Information Technology, Anhui University, Hefei 230601, China;

^6^Songshan Lake Materials Laboratory, Dongguan, Guangdong 523808, China.

^7^These authors contributed equally: Xu Yang and Jia-Wan Li.

* Corresponding authors: [zhcheng@iphy.ac.cn](mailto:zhcheng@iphy.ac.cn) or [houysh@mail.sysu.edu.cn](mailto:houysh@mail.sysu.edu.cn)

**Content**

**S1. The quality of Fe/GeTe and high Bi dopant thin films**

**S2. The ARPES spectrums of Ge_1-x_Bi_x_Te**

**S3. FMR signal fitted by Kittel equation**

**S4. The contribution of the extrinsic factors to the damping**

**S5. k-dependence damping at different Fermi levels**

**S6. Calculated DOS of hybridized states**

**References**

**S1. The quality of Fe/GeTe and high Bi dopant thin films**

Figure S1(a) shows the RHEED pattern and low-energy electron diffraction (LEED) spots of Fe thin film. The LEED pattern of Fe has three bright and three dark diffraction spots, proving the surface of Fe thin film is Fe (111). Combined with Figure 1(c) in the main text, the epitaxy relation of the bilayer is $Fe\langle\overline{1}10\rangle||\text{GBT}\langle\overline{1}100\rangle||\text{Si}\langle\overline{2}11\rangle$. To clarify the quality of the Fe layer on GBT, Figure S1(b) is an HRTEM picture near the Fe and GeTe intersection. It shows the epitaxy interface between Fe and GeTe, and the lattice of Fe can be observed clearly. It means the good quality of Fe thin film on GeTe layer.

**
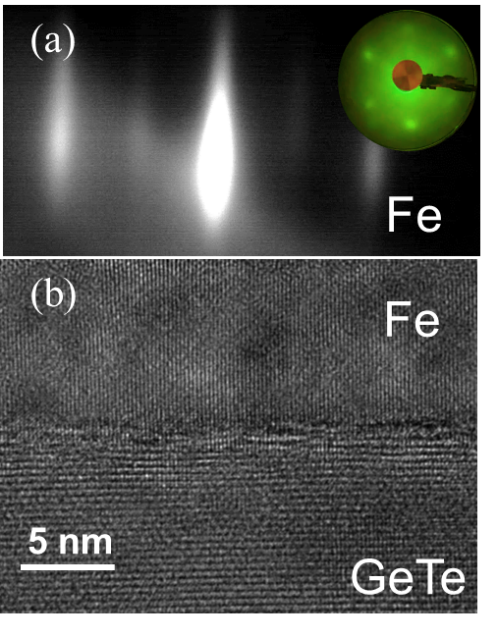
**

**Figure. S1|** **Structure of Fe/GeTe thin film. (a)** RHEED and LEED patterns of Fe thin film. **(b).** HRTEM image of Fe and $\alpha$-GeTe bilayer.

We use the HRTEM to character the quality of the highest composition sample Ge_0.93_Bi_0.07_Te and also analyze the element distribution (shown in Figure S2). The uniform distribution of the Bi element shows that the Bi dopant does not form clusters. The XRD (shown in the main text Figure 1(b))and the HRTEM results imply the Bi elements don’t form other phases, such as Bi_2_Te_3._


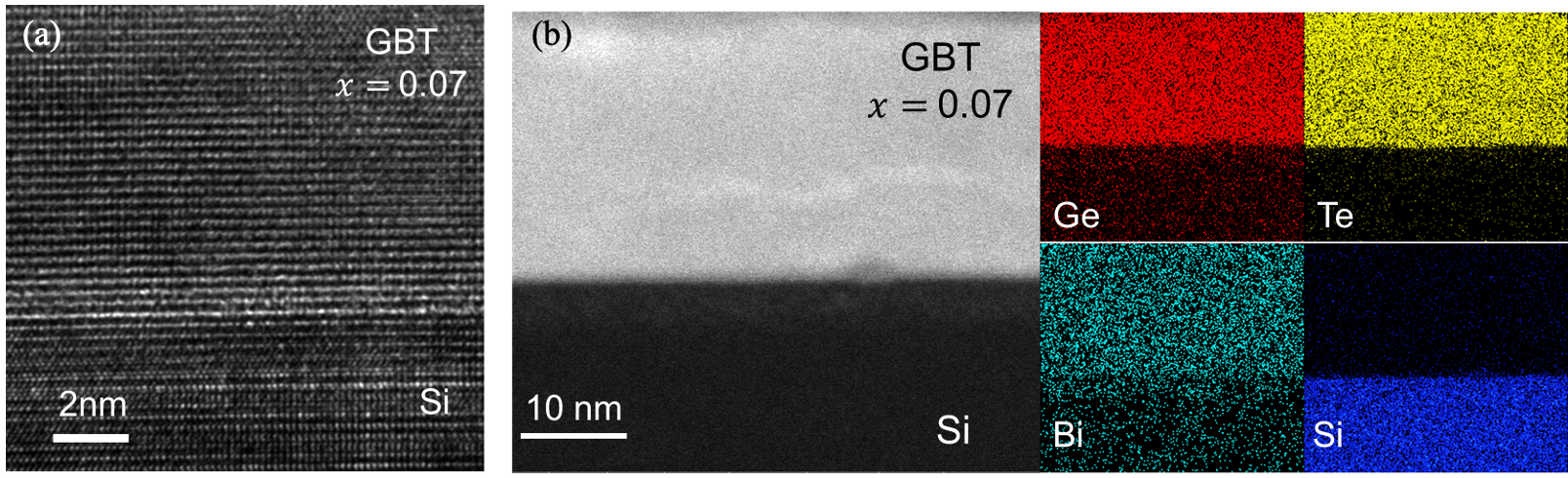


Figure S2 The HRTEM and the high-angle annular dark field (HAADF) images of Ge_0.93_Bi_0.07_Te/Si sample. (a) the HRTEM image of Ge_0.93_Bi_0.07_Te/Si. (b) The HAADF image of Ge_0.93_Bi_0.07_Te/Si cross section and the elemental compositions of Ge,Te,Bi and Si are mapped by red, yellow, cyan and blue, respectively.

**S2. The ARPES spectrums of Ge_1-x_Bi_x_Te**


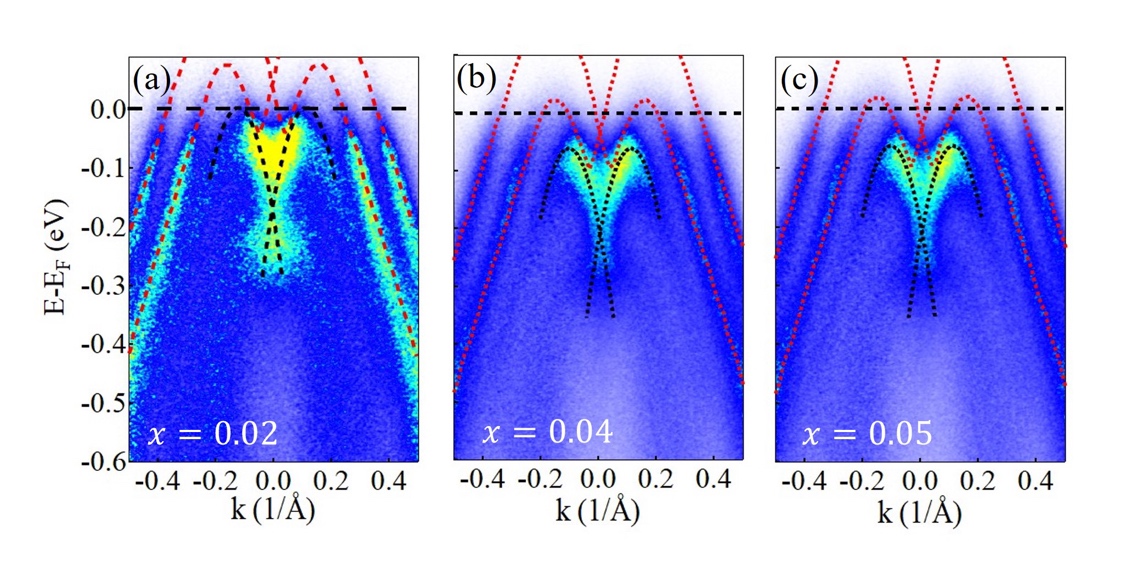


**Figure S3 The ARPES results of Bi-doped GeTe. (a), (b)** and **(c)** are x = 0.02, x = 0.04 and x = 0.05, respectively. The black and red dashed lines are the bulk states and surface states.

**S3. FMR signal fitted by Kittel equation**

Figure S3 presents the frequency dependence of resonance fields $H_{\mathrm{res}}$ for all Fe/GBT samples and the Fe reference sample. The $H_{\mathrm{res}}$ is fitted by the equation S1[1],

$$\begin{aligned} \mathrm{Re}S_{21}\left( H \right)=S_{0}+L\frac{\Delta H^{2}}{\left( H-H_{\mathrm{res}} \right)^{2}+\Delta H^{2}}-D\frac{\Delta H\left( H-H_{\mathrm{res}} \right)}{\left( H-H_{\mathrm{res}} \right)^{2}+\Delta H^{2}}\#\left( S1 \right) \end{aligned}$$

Here $\mathrm{Re}S_{21}$ is the real part of the transmission parameter, $S_{0}$ is the offset, $H$ is the applied magnetic field, $L$ and $D$ are the symmetric and antisymmetric magnitude, respectively. The best fitting results are shown in Figure S4. Then the fitting parameters include $4\pi M_{\mathrm{eff}}$ and $H_{\mathrm{int}}$ are shown in Figure 3(b) in the main text.

Figure S5(a) shows the S_21_ spectrum of all samples at 12 GHz, which can be fitted by a single Lorentzian function. The best fitting results of resonance fields $H_{\mathrm{res}}$ are listed in Figure S5(b). The non-monotonic behavior implies the effective saturation magnetization of Fe varies with Bi concentration due to the interlayer coupling.


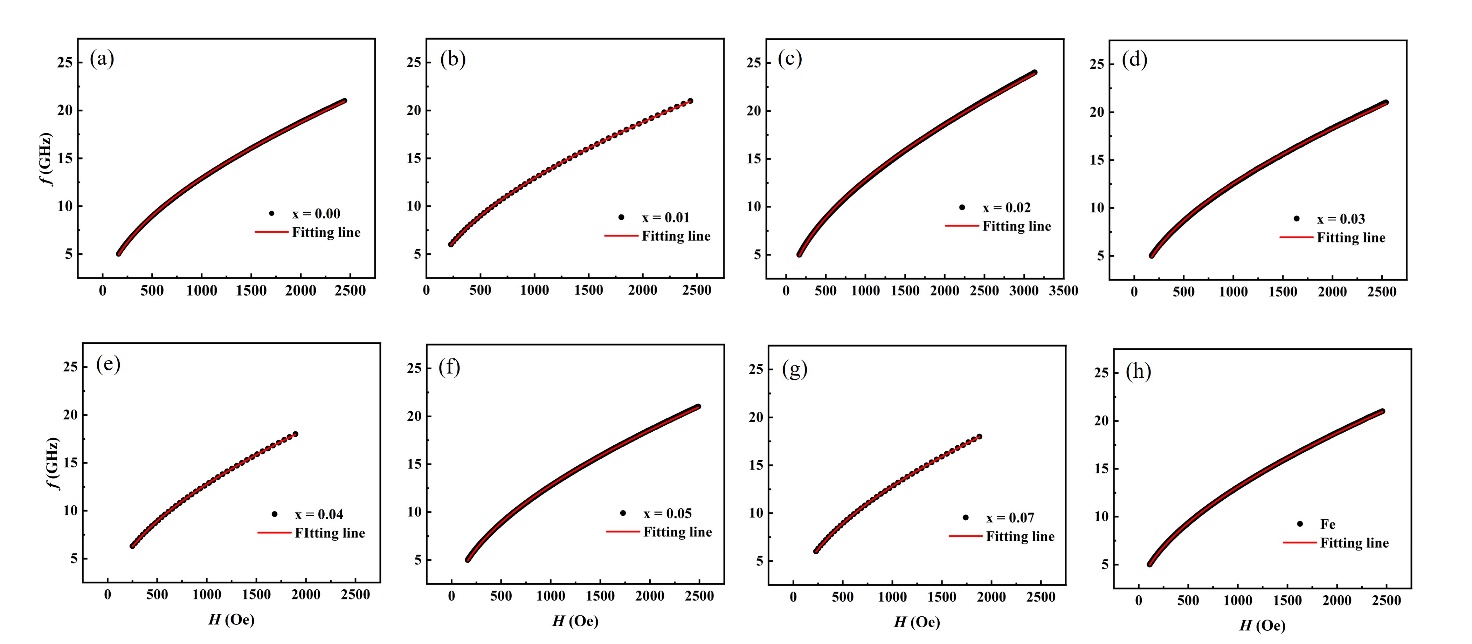


**Figure S4|** **The frequency dependence of resonance fields. (a)-(g).** The function of $H_{res}$ with frequency for Fe/GBT bilayers. The red lines are the fitting lines using the Kittel function in the main text. **(h).** The function of $H_{res}$ with frequency for Fe reference sample.


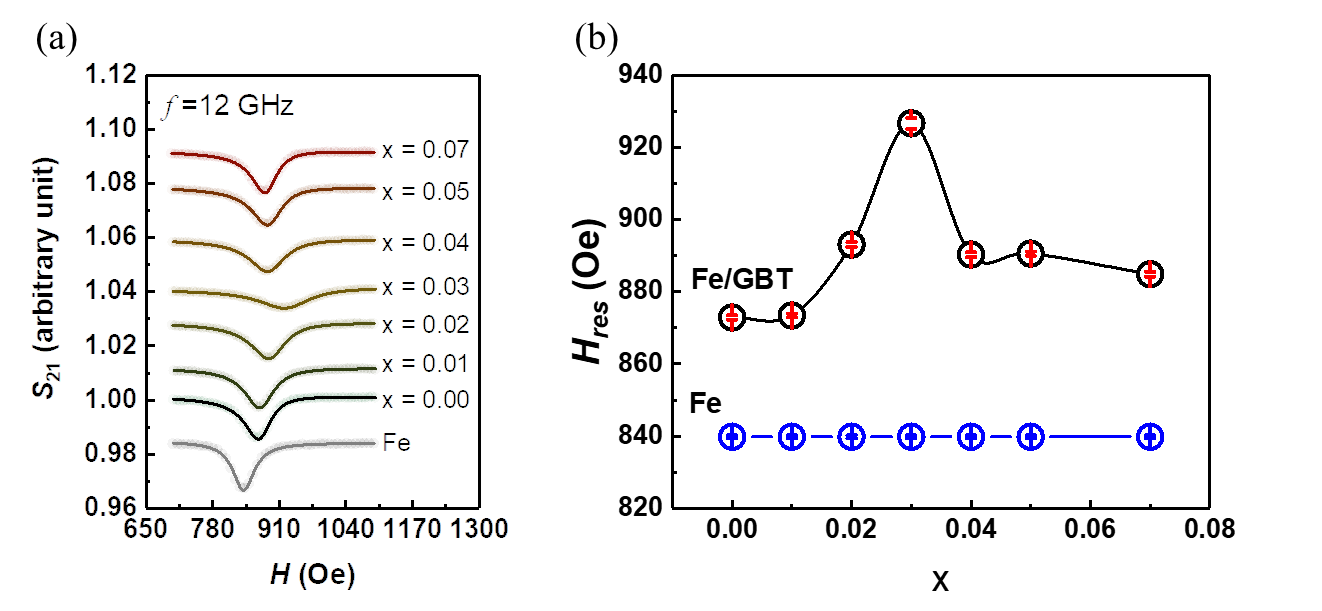


**Figure S5|** **The resonance fields with Bi concentration. a.** Normalized FMR signal S_21_ at 12 GHz for Fe/GBT samples with different Bi concentration and Fe reference sample. **b.** Bi concentration (x) or Fermi level dependence of the resonance fields $H_{\mathrm{res}}$ at 12 GHz extracted from **a** fitting by Lorentz function. The blue line represents the resonance fields of Fe reference sample at12 GHz. **c.** Frequency dependence of FMR linewidth for all Fe/GBT samples and Fe reference thin film.

**S4.** The contribution of the extrinsic factors to the damping

It is well-accepted in the community that the contribution of extrinsic factors to the damping would lead to a non-linear relationship between the FMR linewidth and resonant frequency, especially the two-magnon scattering. This is because the influence of two-magnon scattering contribution becomes smaller at higher magnetic fields when larger microwave resonance frequencies are applied. It is a natural scenario to investigate the FMR spectra at higher microwave frequencies to reveal the presence of the two-magnon scattering. The interfacial quality plays a key role in two-magnon or multi-magnon scattering.

In order to precisely determine the Gilbert damping, α, and zero-frequency linewidth $\Delta H_{0}$, as well as taking the effect of two-magnon scattering into account, we fitted the experimental $\Delta H$ versus *f* using the equation including the two-magnon scattering[2]:

$$\begin{aligned} \Delta H=\Delta H_{G}+\Delta H_{2M}+\Delta H_{0}\#\left( S2 \right) \end{aligned}$$

Here, $\Delta H_{G}=\frac{4\pi\alpha f}{\mu_{0}\gamma}$ represents the linewidth contribution from the Gilbert damping term. $\Delta H_{2M}=\Gamma\sin^{-1} \sqrt{\frac{\sqrt{\left( 2\pi f \right)^{2}+\left( \frac{2\pi f_{0}}{2} \right)^{2}}-\left( \frac{2\pi f_{0}}{2} \right)}{\sqrt{\left( 2\pi f \right)^{2}+\left( \frac{2\pi f_{0}}{2} \right)^{2}}+\left( \frac{2\pi f_{0}}{2} \right)}}$ is the linewidth contribution from the two-magnon scattering with $f_{0}=\frac{\gamma\mu_{0}}{2\pi}M_{eff}$ and the strength of two-magnon scattering $\Gamma$.

Firstly, we found that the inhomogeneous linewidth broadening (i.e., zero-frequency offset, $\Delta H_{0}$) is much smaller (~ 3 Oe) compared with the overall damping, suggesting the high-quality interface of our epitaxial single crystal films (shown in Figure S6).


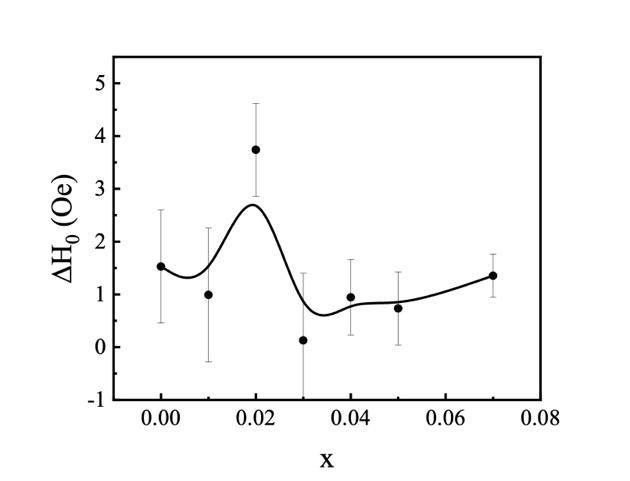


Figure S6 The zero-frequency linewidth $\Delta H_{0}$ with different Bi concentrations.

Secondly, the two-magnon scattering should result in a nonlinear $\Delta H$ versus *f*. The results, shown in the main text Figure 3(c), can be best fitted linearly with R^2^～0.99 at different Bi concentration samples. Such a linear frequency dependence of linewidth persisting throughout the frequency range provides a unambiguous evidence that the contribution of two magnon scattering to the linewidths does not account for the observed damping. As shown in Figure S7, the contribution of two-magnon scattering is rather negligible (the maximum contribution of two-magnon scattering to the FMR linewidth is smaller than 1 Oe at 16 GHz for all samples, which is less than 1% ($\Delta H_{2M}/\Delta H_{total}$) of the total FMR linewidth.). Thus we concluded that $\Delta H$ versus *f* is predominantly affected by the Gilbert contribution in our high-quality films.


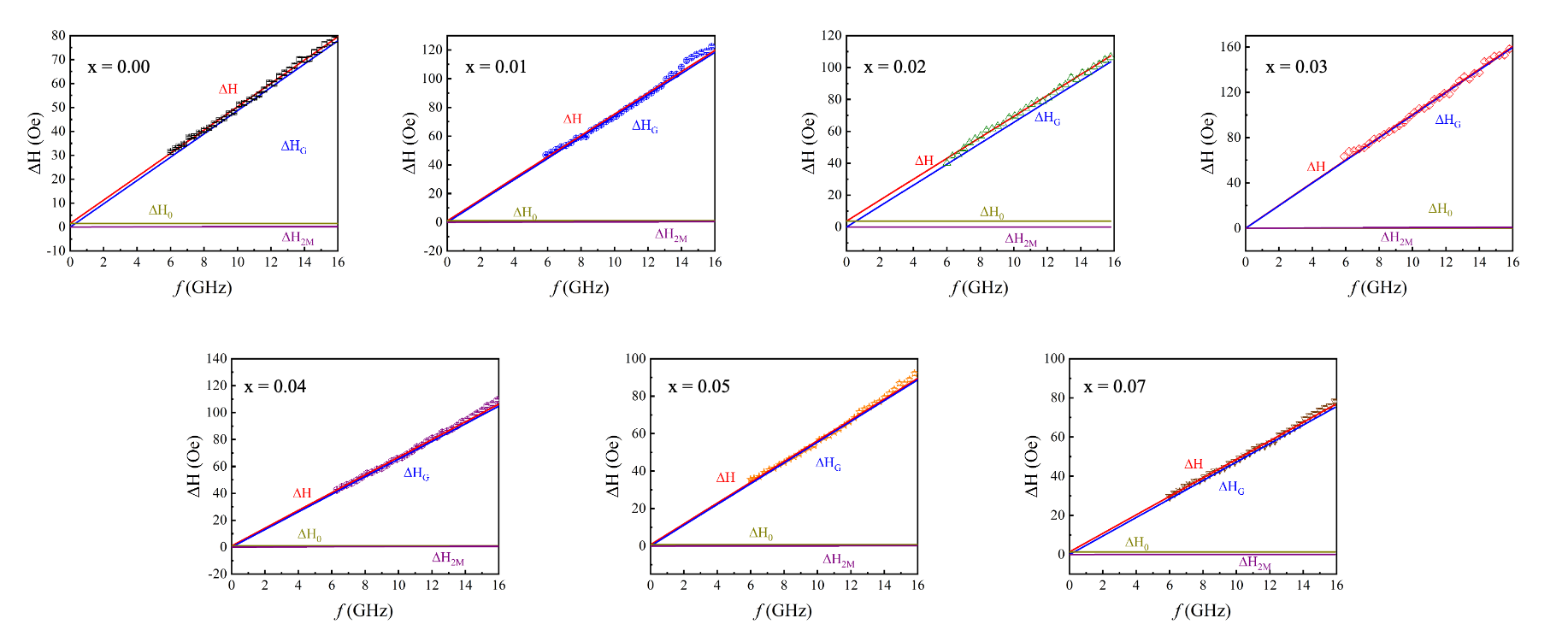


Figure S7 The relationship between resonant linewidth ΔH and resonant frequency *f* of all Fe/Ge_1-x_Bi_x_Te samples fitted by equation *S*2.

**S5. k-dependence damping at different Fermi levels**

The magnetic damping calculation by DFT does not consider the spin-pumping effect. The DFT results only considered the influence of band hybridization between Fe and GeTe. We use this method to calculate the magnetic damping because the bulk states of GeTe are far away from the Fermi level with increasing the Bi concentration, and the surface states do not impact the spin-dependent phenomena because of the strong hybridization between Fe and GeTe. This means the spin-pumping effect is weaker and weaker as increasing the Bi concentration. Therefore, the spin-pumping effect does not play a key role in our experiment. The hybridization is a key factor to influence the magnetic damping, and we give the k dependence damping at different Fermi levels shown in the Figure S8. When the Fermi level is $-0.15$ eV, the contributions to Gilbert damping are almost from the *k* points in the vicinity of the $\Gamma$ point where the surface Rashba state appears in the free-standing GeTe film. In contrast, when the Fermi level deviates from $-0.15$ eV, large contributions to Gilbert damping can come from random *k* points in the first BZ. Comparing these random k points with the band structures, all the bands at these random *k* points are the hybridized bands.

**S6. Calculated DOS of hybridized states**

Figure S9 is the calculated DOS of hybridized states at different regions. We calculated three regions: the whole BZ, the quadrilateral region, and the hexagonal region, signed by (1), (2), and (3). To figure out the influence of the Fe spectrum weight and GeTe spectrum weight, we calculated the ratio DOS of $\frac{N_{\mathrm{Fe}}}{N_{\mathrm{Fe}}+N_{\mathrm{GeTe}}}$ and $\frac{N_{\mathrm{GeTe}}}{N_{\mathrm{Fe}}+N_{\mathrm{GeTe}}}$ at different regions (shown in Figure S9 (b)-(f) and Figure 4(c) in the main text), separately. We found that only in the hexagonal region, the $\frac{N_{\mathrm{GeTe}}}{N_{\mathrm{Fe}}+N_{\mathrm{GeTe}}}$ has the similar behavior with the damping. Therefore, we can conclude that the damping of the Fe/GeTe verified by the Fermi level is related to the DOS of GeTe spectrum weight of the hybridized states.


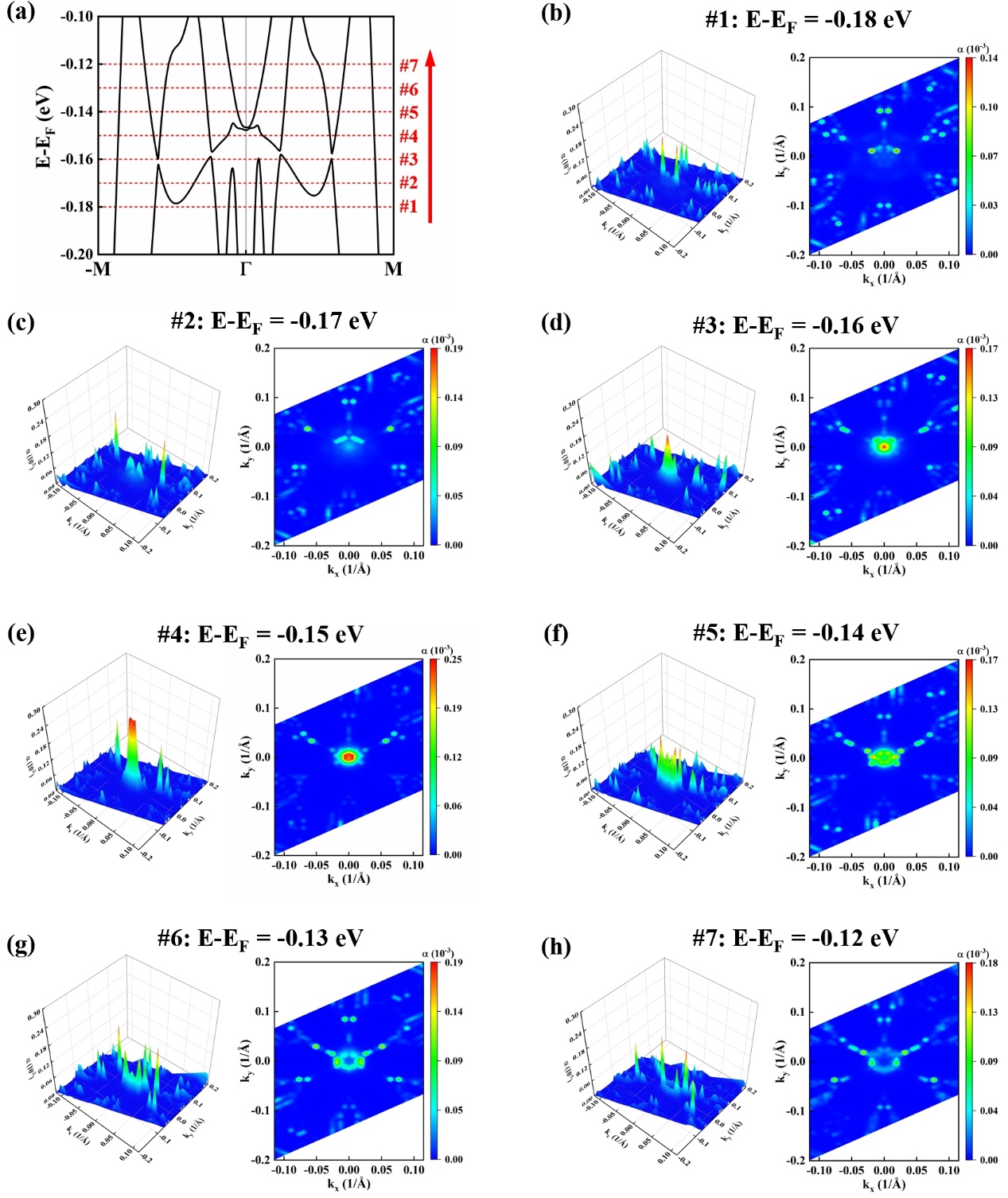


**Figure S8 | The k-dependence damping at different Fermi levels**. **(a)** Band structure of Fe/GeTe. The red horizontal lines indicate the energy level indexed by #*i* (*i*=1,2,…,7) for **(b)-(h)**. The first BZ distributions of *k*-dependent contributions to Gilbert damping with different E-E_F_ are shown in **(b)-(h)**.


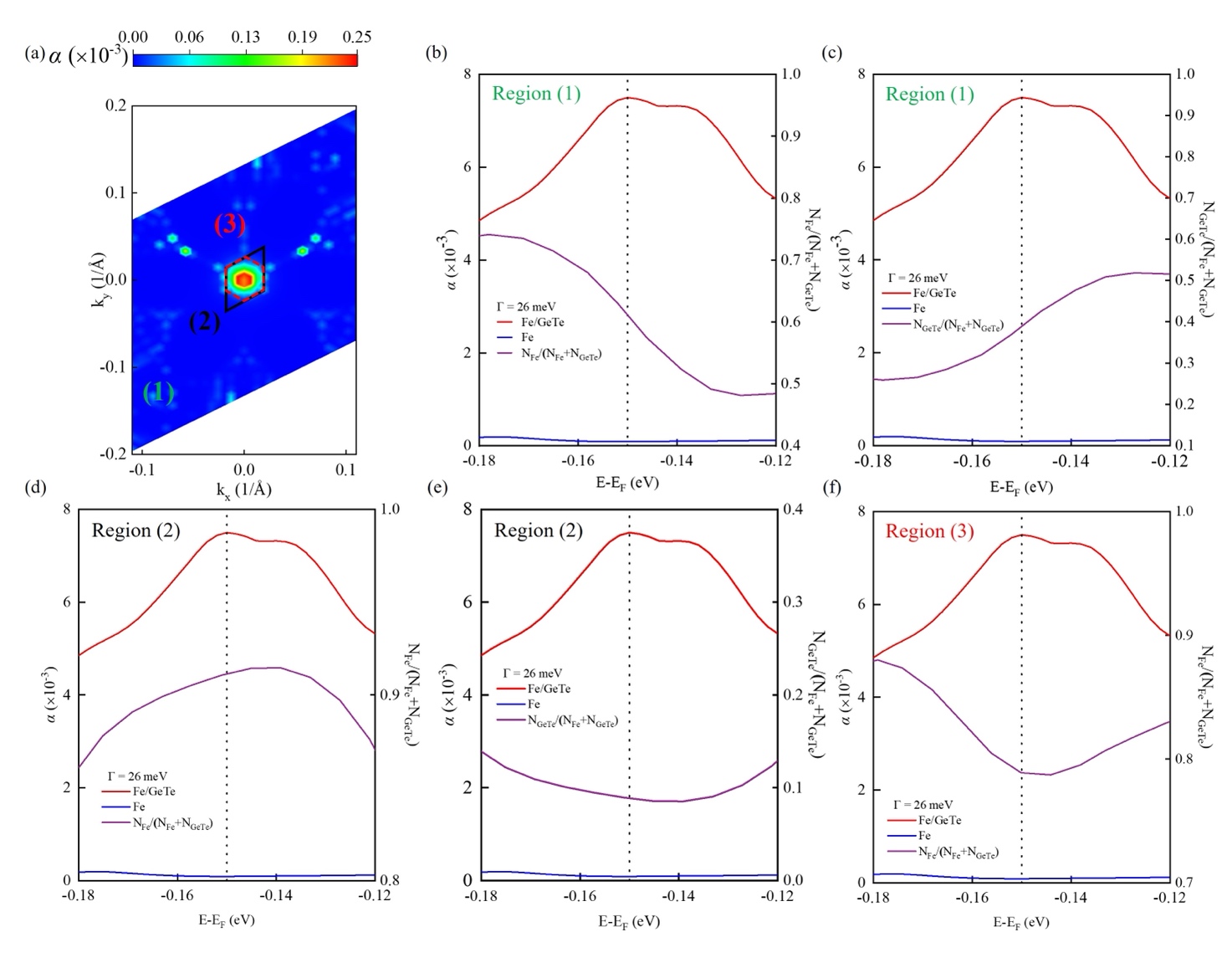


**Figure S9 | The DOS of hybridized bands at different BZ regions**. **(a)** the k-dependence damping at $E-E_{F}=-0.15 \mathrm{eV}$, and the three calculated regions are signed by (1) the whole BZ, (2) the quadrilateral region, and (3) the hexagonal region. **(b)-(f)** are the ratio DOS of $\frac{N_{\mathrm{Fe}}}{N_{\mathrm{Fe}}+N_{\mathrm{GeTe}}}$ and $\frac{N_{\mathrm{GeTe}}}{N_{\mathrm{Fe}}+N_{\mathrm{GeTe}}}$ at different regions.

**References**

[1] S. S. Kalarickal, P. Krivosik, M. Wu, C. E. Patton, M. L. Schneider, P. Kabos, T. J. Silva, J. P. Nibarger, *J. Appl. Phys.* **2006**, *99*, 093909.

[2] a) R. Arias, D. L. Mills, *Phys. Rev. B* **1999**, *60*, 7395; b) R. Arias, D. L. Mills, *J. Appl. Phys.* **2000**, *87*, 5455; c) K. Lenz, H. Wende, W. Kuch, K. Baberschke, K. Nagy, A. Janossy, *Phys. Rev. B* **2006**, *73*, 144424.
